# Tissue Doppler, Strain and Strain Rate in ischemic heart disease “How I do it”

**DOI:** 10.1186/1476-7120-12-38

**Published:** 2014-09-18

**Authors:** Razvan O Mada, Jürgen Duchenne, Jens-Uwe Voigt

**Affiliations:** Department of Cardiovascular Diseases, University Hospital Gasthuisberg, Catholic University Leuven, Herestraat 49, 3000 Leuven, Belgium

**Keywords:** Echocardiography, Deformation imaging, Tissue Doppler, Speckle tracking, Strain, Strain rate, Myocardial ischemia

## Abstract

Echocardiography is the standard method for assessing myocardial function in patients with ischemic heart disease. The acquisition and interpretation of echocardiographic images, however, remains a highly specialized task which often relies entirely on the subjective visual assessment of the reader and requires therefore, particular training and expertise. Myocardial deformation imaging allows quantifying myocardial function far beyond what can be done with sole visual assessment. It can improve the interpretation of regional dysfunction and offers sensitive markers of induced ischemia which can be used for stress tests. In the following, we recapitulate shortly the pathophysiological and technical basics and explain in a practical manner how we use this technique in investigating patients with ischemic heart disease.

## Background

Assessing the impact of ischemic heart disease on cardiac function is one of the key tasks of routine clinical echocardiography. At virtually any stage of the disease process, echocardiography is of diagnostic or prognostic value, guiding further therapeutic decisions. The careful observation of global and regional ventricular function and morphology in resting images, allows describing and distinguishing acute ischemia, scar and infarct related remodeling while stress echocardiographic methods may be used to assess the severity of coronary stenosis or to identify viable myocardium. Offering this wealth of information, echocardiography has become an indispensable tool in managing patients with coronary artery disease.

The acquisition and interpretation of echocardiographic images, however, remains a highly specialized task which requires knowledge, skills and experience. Not all echocardiographic parameters are quantifiable. Moreover, typical echocardiographic tasks, such as the evaluation of regional myocardial dysfunction in ischemic heart disease, rely entirely on the subjective assessment of the reader and require therefore particular training and expertise [1–4].

Tissue Doppler and speckle tracking based myocardial deformation imaging allows quantifying myocardial function far beyond what can be done with sole visual assessment. In the following, we would like to explain how we use this technique in investigating patients with ischemic heart disease.

### How to measure myocardial function

Information on the technical and basic clinical applications of echocardiographic deformation imaging may be found elsewhere [5–7]. In short, regional myocardial function can be assessed by measuring strain or strain rate. Velocity and displacement are not suited for this task. Strain is defined as the fractional change in length of a myocardial segment relative to a baseline length and is expressed as percentage. Strain rate is the temporal derivative of strain and provides information on the speed at which the deformation occurs, expressed as [sec^−1^]. In clinical use, the complex three-dimensional deformation of the heart is commonly described by either its longitudinal (LS), circumferential (CS) or radial strain (RS) components (“normal strain components”). There are two main echocardiographic modalities for the assessment of myocardial deformation: tissue Doppler imaging (TDI) or speckle-tracking imaging (STI) (see also Tables 1 and 2).

**Table 1:** **Comparison of recommended steps for tissue Doppler and speckle tracking based deformation imaging**

	Tissue Doppler	Speckle tracking
**Image acquisition**	- TDI images with insonation angle <15° (single wall acquisition if needed) and adequate velocity scale (to avoid aliasing)	- Optimized 2D images avoiding foreshortening and stationery artefacts (reverberation)
	-Frame rate > 100 fps (ideally > 140 fps)	- Frame rate: 40–80 fps
	- Acquire spectral Doppler traces of mitral and aortic valve for timing	- Acquire a spectral Doppler trace at least of the aortic valve as backup for timing
**Image analysis**	- Measure aortic and mitral valve opening and closure in order to define cardiac phases	
	-Order of image analysis: arbitrary	- Start analysis with the apical long axis view in order to define aortic valve closure. Use Doppler derived data if needed.
	- Set the region of interest (ROI) shape and size oval somewhat smaller than the wall thickness. Position the needed number of ROIs it in the middle of the segments to be analysed. Track ROI position if needed.	- Contour the myocardium according to the procedure proposed by the vendor. Avoid including the bright pericardium.
	- Evaluation of curve quality. Repositioning of the ROI if needed.	- Careful visual control of the proposed tracking. Repositioning of the contours if needed. Exclusion of suboptimally tracked segments if re-contouring is unsuccessful.
	- Shape analysis of the curves	- Shape analysis of the curves
	- Measurement of peak values and further post-processing if needed	- Measurement of peak values and further post-processing if needed

**Table 2:** **Advantages and limitations of tissue Doppler and speckle tracking based deformation imaging**

	Advantages	Limitations
**Tissue Doppler**	- High temporal resolution	- Dedicated image acquisition needed.
	- Robust in case of limited image quality	- One dimensional: measures deformation along the ultrasound beam direction
	- Easy recognition of artefacts, easy assessment of data quality	
		- Noisy data, sensitive to stationary artefacts
	- Fast qualitative analysis: curves readily available by moving the mouse pointer over the myocardium (no post- processing needed)	
		- Region of interest must be tracked to keep the same position within the myocardium
		- Training needed
**Speckle tracking**	- Standard 2D images used for analysis	- Lower temporal resolution
		- Strong dependence on image quality
	- Two-dimensional analysis possible	
		- Intensive regularization and drift correction with limited options for user interaction
	- Less noisy data	
	- Very user friendly	
		- Difficult assessment of data quality
	- Easy post-processing, wealth of graphical displays	
		- Intervendor differences in parameter definitions and measurement algorithms
	- Highly automated extraction of derived parameters	
		- Need for training underestimated

### Tissue Doppler derived deformation

TDI is the classic approach to measure deformation in echocardiographic images [8]. From TDI data, strain rate can be simply derived by calculating the regional velocity gradient. By integration of the latter, strain is obtained. Major advantages of TDI derived strain measurements are the very high temporal resolution and the relative robustness if image quality is limited. After some training, curves are easy to interpret and allow a good distinction between good quality data and artefacts. For simple curve shape analysis in clinical use, no post-processing is needed since curves are readily available by just moving the mouse pointer over the myocardium. Disadvantages comprise the angle dependency of the data and the need for training and experience when advanced data handling and interpretation are needed.

### Speckle tracking derived deformation

**Figure 1:**
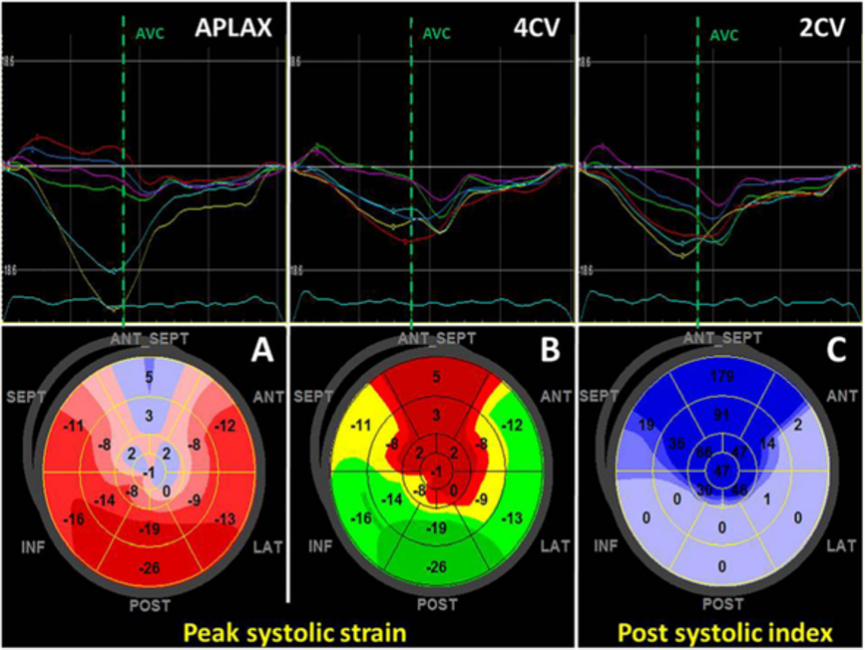
**Speckle tracking based strain analysis in a patient with myocardial infarction.** *Upper panels:* physiologic and pathologic segmental strain curves from the three apical standard views. *Lower panels:* bull’s eye displays of the regional longitudinal strain; **A** and **B**, peak systolic strain in two different colour maps; **C**, post systolic index. Note the clear delineation of the anteroseptal infarct area. AVC - aortic valve closure; APLAX, 4CV, 2CV – apical long axis, four- and two-chamber view.

STI is a newer approach to assess myocardial deformation [9]. It relies on detecting features (mostly “speckles”) in 2D greyscale images the displacement of which is measured. Velocity, strain and strain rate is then derived from the displacement information. Speckles can be tracked in any direction within the image plane which offers new options for motion and deformation analysis compared to the one-dimensional TDI method. STI depends strongly on good image geometry and quality, both of which are often difficult to achieve in routine clinical practice. The need for good spatial resolution (i.e. high line density) limits the frame rate at which grey scale data can be acquired, leading to a lower temporal resolution of STI data. Modern analysis software offer many display options for tracking derived parameters. Besides the classic display of colour overlays, curves and curved M-modes, tracking results from different image planes can be combined and displayed in a bull’s eye format which allows a fast and easy assessment of any tracking derived parameter (Figure 1) [10]. Currently, global longitudinal strain (GLS) emerges as new routine measure of ventricular function. It has been shown to be a robust and reproducible parameter, superior to classical ejection fraction (EF) measurement, probably due to the largely automated assessment and extensive spatial averaging. Inter-vendor differences in strain measurements must be considered. A joint task force of EACVI, ASE and Industry has recently prepared a consensus document for standardized STI based deformation imaging [11].

### Which function changes can we expect?

#### Load dependence

Functional imaging shows motion and deformation of the myocardium within the image plane. Any change in perfusion or the underlying metabolism of the myocardial fibres will remain invisible unless it affects their contractile state. Further, imaging cannot measure pressures or forces and is, thus, “blind” to loading conditions. Any observed myocardial deformation is consequently a product of the contractile state of the myocardial fibres, modulated by pre- and afterload. It is therefore important to understand, that all findings in functional cardiac imaging do not directly relate to myocardial contractility, but must be interpreted in the context of wall thickness, chamber geometry and loading conditions.

### Ischemic function changes

The imbalance between oxygen demand and supply results in an impairment or loss of contractile function and, subsequently, cell death. Transient ischemia will cause reversible regional dysfunction. Even without structural damage, however, the dysfunction may last for hours after an ischemia which is called “stunning” [12, 13]. A chronic reduction of coronary flow leads to a proportional myocardial dysfunction named “hibernation”. Hibernating myocardium may also recover completely after restoration of blood flow, but degenerative changes and persistent dysfunction may occur after longer malperfusion [14]. Coronary occlusion lasting more than a few minutes results in irreversible myocardial injury with later scar formation [15]. Cardiac remodeling will occur depending on the amount of myocardial loss [16, 17].

The particular anatomy of the coronary arteries and the higher extravascular pressure make the subendocardial region most vulnerable to ischemia. Dysfunction will therefore firstly occur in the subendocardial fibres [18]. To which extend layer-specific (dys-)function can be evaluated by echocardiography, remains to be determined.

Functional imaging is blind to stenosis of the epicardial vessels unless regional dysfunction develops due to ischemia. Pharmacological, physical or electrical stress tests are needed to induce such ischemia and belong, therefore, to the standard armamentarium of echocardiography in the clinical setting of ischemic heart disease [4, 19, 20].

### Characteristic deformation patterns in disease

Two principal changes can be observed with deformation imaging.

Type 1: The reduction in systolic strain indicates myocardial dysfunction, but may also be attributed to changes in loading conditions or ventricular geometry. The reliable detection of abnormal strain amplitudes requires a robust and reproducible measurement method. Under clinical conditions, the variability of both regional tissue Doppler and speckle tracking measurements is relatively high, which makes regional strain amplitude measurements currently less suited for routine use. This is in contrast to global strain measurements which have been shown to be reproducible and robust measures of global ventricular function, superior to classical EF measurements, probably due to the largely automated assessment and extensive spatial averaging [21].

**Figure 2:**
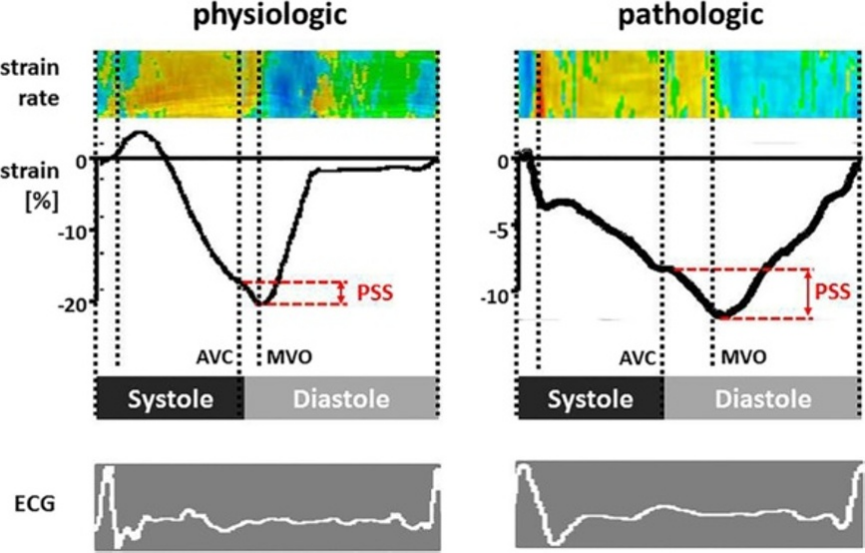
**Colour coded strain rate curved M-modes (top) and strain curves (middle) in 2 myocardial segments both showing post-systolic shortening (PSS).** *Left panel:* physiologic PSS in an apical septum of a normal heart. Peak systolic strain exceeds −18% and PSS is less than 20% of systolic shortening. *Right panel:* pathologic PSS in a partially scarred segment post infarction. Peak systolic strain is reduced to −10.1% while post systolic strain is increased to 39% of systolic strain. *AVC* - Aortic valve closure; *MVO* - mitral valve opening. (modified from Voigt et al. [22], with permission).

Type 2: Regional inhomogeneities of myocardial activation and/or contractility can alter the temporal sequence of shortening and lengthening of the myocardium. In ischemia, not only the amplitude of shortening is reduced, but also onset and duration of fibre contraction are altered, which leads to a characteristic shortening or thickening of the myocardium after aortic valve closure. This so-called “post-systolic shortening” (PSS) or “post-systolic thickening” is a sensitive and specific feature of developing ischemia if a normal baseline examination is available [22, 23]. If found under resting conditions, it is a sensitive but unspecific sign of regional dysfunction of any cause (scar, dyssynchrony, etc.) [24, 25]. It must be noted, that PSS of a minor extend can be found in normal hearts, particularly at the apex and base of the inferior, septal and anteroseptal walls (Figure 2) [24].

### “How I do it” - Practical approaches in particular situations

#### Chronic ischemic disease

Patients with myocardial damage after myocardial infarction will usually present changes in global and regional function, both of which need to be described and followed. In these patients, a speckle tracking based analysis can be most easily integrated in the clinical routine.

### Data acquisition

Three standard grey scale image loops, with settings balanced for both spatial and temporal resolution (ca. 40–80 frames per second), covering the LV and including the mitral and aortic valve should be stored. Care must be taken that the LV is not foreshortened and that reverberation artefacts (bright, wedge-shaped, stationery artefacts) on the edges of the image sector are avoided. A clear artefact free display of the apex is desired. We acquire three consecutive heart beats, avoiding extrasystoles. In addition, a PW- or CW-Doppler trace of the LVOT is useful to obtain timing information.

### Data analysis

**Figure 3:**
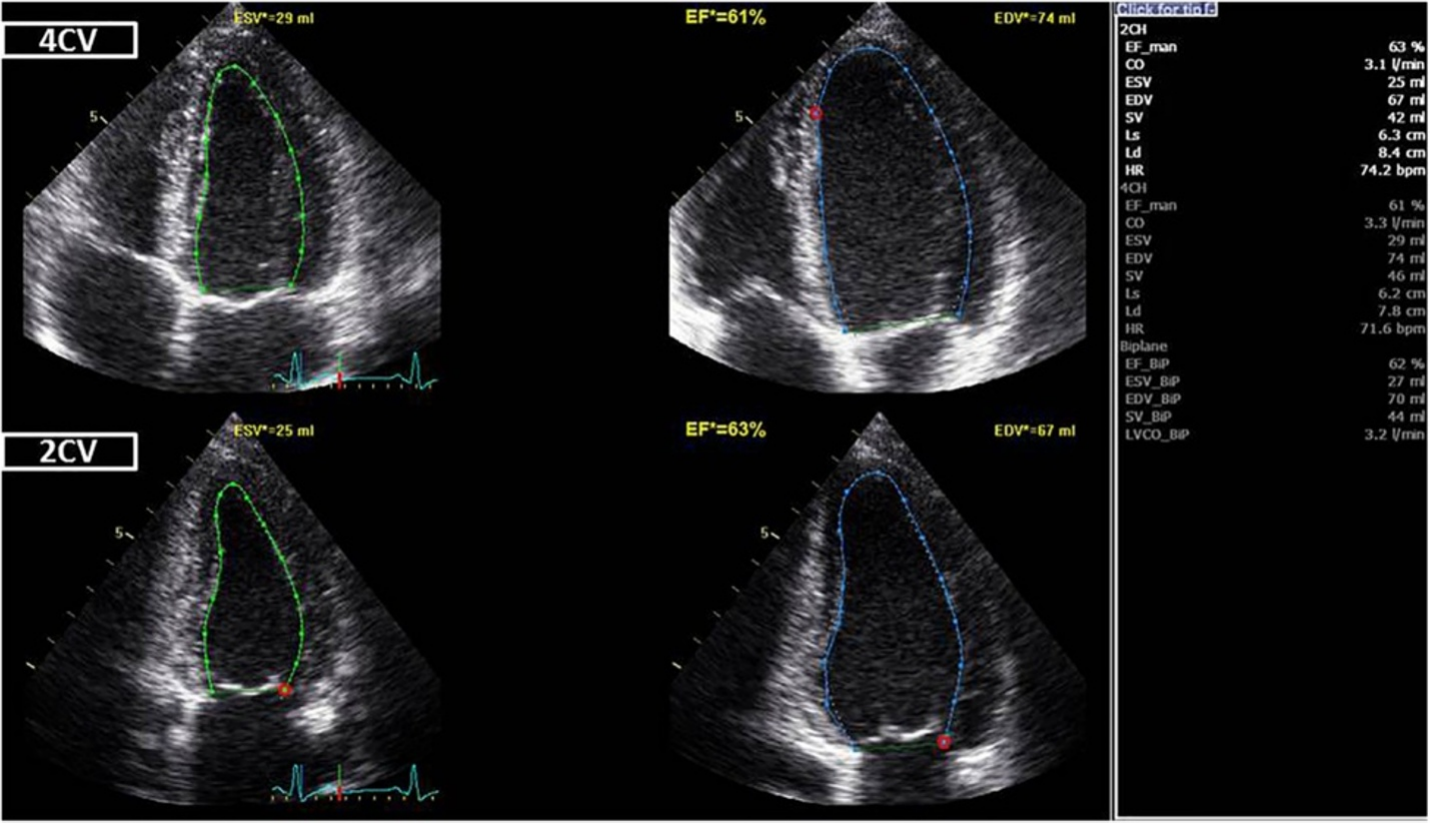
**Tracking based ejection fraction measurement.** Fully automated delineation and tracking of the endocardial contour throughout the cardiac cycle allows a fast and reproducible bi-plane assessment of global LV function and volumes. *EF* - ejection fraction; *CV* - chamber view; *EDV* - end-diastolic volume; *ESV* - end-systolic volume.

First, the four- and two-chamber images are analyzed with an automated, tracking based EF measurement algorithm. Depending on the software used, this can be fully automatic or semi-automatic with the user indicating the initial endocardial contour. Care must be taken to check the tracking quality, i.e. how well the tracking line follows the endocardial motion. Software which do not allow this step should not be used. This procedure results in a set of volumetric measurements (volumes, ejection fraction, stroke volume, cardiac output) which is reported in the protocol of the examination (Figure 3).Secondly, after manually defining aortic valve closure through direct visualisation in the apical three-chamber view or by using spectral Doppler data as alternative, myocardial speckle tracking of all three apical image planes is performed. This is again a fully- or semi-automated process as described above and the quality of tracking needs to be checked in a similar way. The position of the tracking region depends on the vendor specific software and may comprise just the endocardium and epicardium or the full wall. Accordingly, expected strain values will differ. The largest body of evidence exists for full wall strain measurements, but several companies do also/only offer endocardial strain. This analysis delivers GLS as average of all three apical views, which is then reported in our echo protocol. Further, the bull’s eye view of LV systolic strain can be used to support regional function analysis. Special colour maps, e.g. for the post-systolic index, i.e. PSS relative to the peak strain, are particularly sensitive for regional dysfunction (Figure 1).

#### Acute ischemia

This is usually an emergency room scenario where an immediate coronary intervention has highest priority. The echocardiographic examination should therefore be limited to the necessary minimum which is needed to clarify remaining clinical questions. Conclusions will be usually drawn from the immediate visual analysis of the image data.

### Data acquisition and analysis

**Figure 4:**
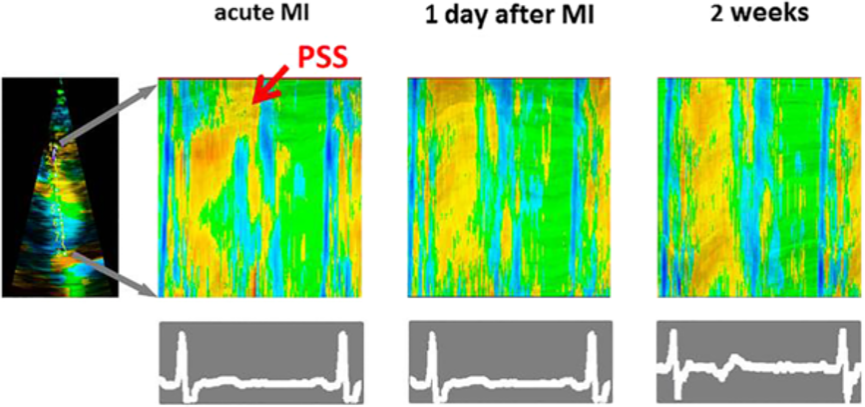
**Curved M-modes of a patient with acute myocardial infarction.** *Left panel*: in the acute phase, a delayed onset of systolic shortening with pathologic post-systolic shortening (PSS) can be noted in the apical segment (arrow). *Middle and left panel*: One day and 2 weeks after the acute coronary intervention the regional function has normalized.

A documentation of the three apical views is a matter of seconds and should be attempted. It allows a later off-line speckle tracking based analysis as described above, which can serve as baseline investigation to follow the course of the patient (Figure 4).

#### Ischemia testing

Ischemia testing aims at detecting transient regional dysfunction during an exercise or pharmacological stress which requires training and experience [1–4]. Functional imaging can help to objectify findings and increase the diagnostic and prognostic value of the test [23, 26, 27]. Stress testing is therefore one of the most important applications of functional imaging.

### Data acquisition

Tissue Doppler strain is robust for shape analysis, quickly analysable during data review and can easily deal with high heart rates due to its excellent temporal resolution. It is therefore our preferred method during stress echocardiography. We use a machine which allows the simultaneous acquisition of grey scale and colour tissue Doppler data while only the grey scale image is displayed. Care must be taken that the underlying colour Doppler sector has the size of the full image, while the frame rate should not fall far below 100 fps. We acquire apical four-, three- and two-chamber views as they allow to measure longitudinal strain in all segments. We perform stress echos according to current recommendations [19]. Dobutamine is the preferred stressor since it allows convenient image acquisition over three consecutive cycles during a breath hold even at peak stress. Bicycle stress is also possible, but makes data acquisition more challenging. As in regular stress echo examinations, the acquisition of identical image planes at each stage is crucial.

### Data analysis

**Figure 5:**
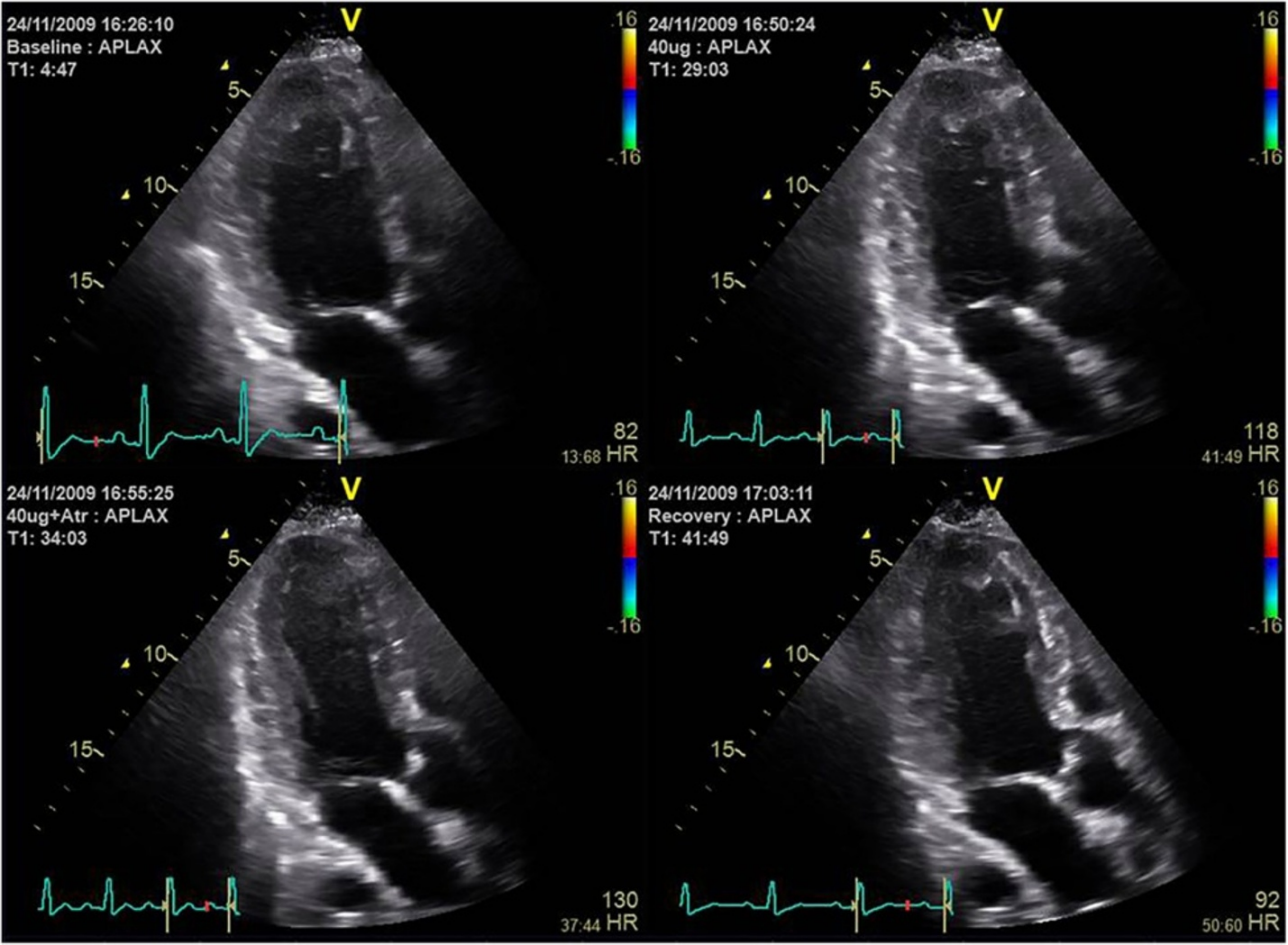
**For the optimal visual assessment of wall motion in stress echocardiograms, a synchronized replay of the different stress stages of the examination in a quad-screen display is crucial.** Note the colour bars in the image which indicate that colour tissue Doppler data have been acquired simultaneously which allow the quantification of myocardial function.

**Figure 6:**
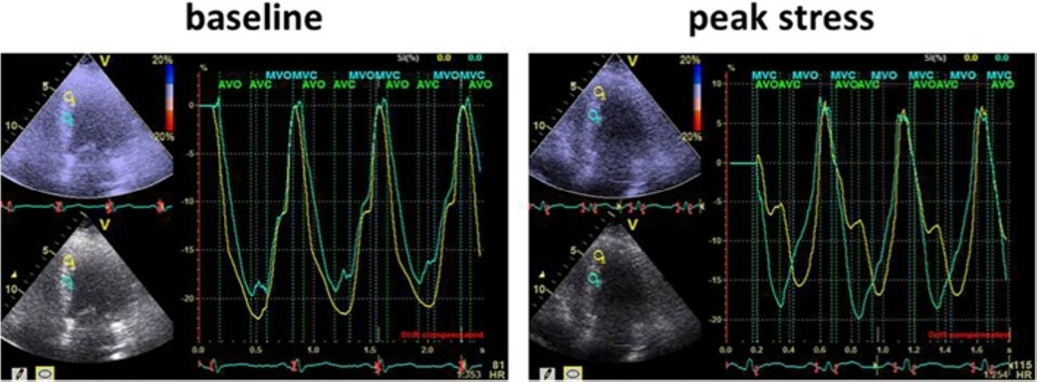
**If there is any doubt about regional function, tissue Doppler strain is the fastest means of regional function analysis in a stress echocardiogram.** *Right panel:* the yellow region of interest shows reduced systolic strain and marked post-systolic shortening at peak stress, suggesting inducible ischemia. This becomes particularly obvious if compared to the green region of interest which shows a normal strain curve. Note that the overall strain amplitude is similar in both segments. *Left panel:* inducible ischemia is distinguished from a pre-existing abnormality by demonstrating that the yellow region of interest shows a normal strain curve at rest. *AVO, AVC, MVO, MVC* – aortic and mitral valve opening and closure.

**Figure 7:**
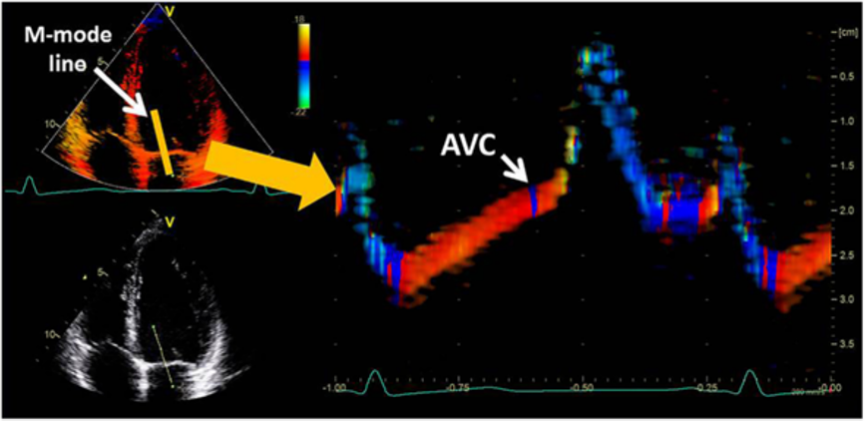
**Timing of aortic valve closure (AVC) can be estimated from a colour M-mode across a mitral leaflet (yellow line).** Note the small blue line at the systolic upstroke of the mitral leaflet contour, the beginning of which indicates aortic valve closure.

**Figure 8:**
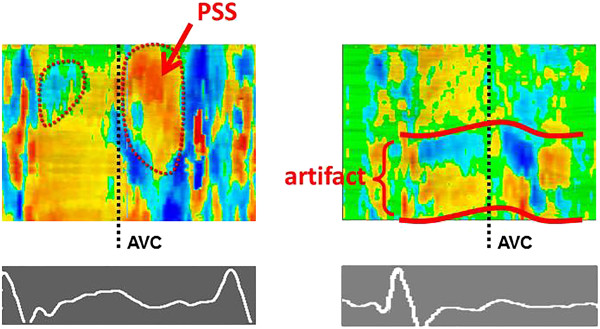
**Distinguishing regional dysfunction and artefacts.** *Left panel:* Regional ischemia is characterized by local changes in systole and early diastole and presents usually with a delayed onset of systolic shortening with marked post-systolic shortening (PSS) (here: apical segment). *Right panel:* Reverberation artefacts results in a typical band-like pattern of inverted colours around the position of the artefact which extends throughout the cardiac cycle.

The classical visual assessment of wall motion in a quad-screen display of baseline and different stress stages with synchronized replay is our standard approach (Figure 5). In case of any doubt, regional strain is analyzed. This is possible “on the fly” since the underlying tissue Doppler data allow an immediate conversion into a strain map and the direct display of regional strain curves. By moving the mouse pointer over the myocardial wall, the changing shape of the strain curves is assessed with a particular focus on the detection of a relevant amount of PSS [23]. In most cases, a rough estimate of end-systole can be obtained by simultaneously showing a strain curve from a normal wall (Figure 6). If this appears not sufficiently accurate, aortic valve closure is measured in the three-chamber view directly or via the colour Doppler display of the anterior mitral valve leaflet as a reference (Figure 7) [24]. In all segments where PSS is detected at peak stress, baseline images are examined in the same way to confirm a normal strain pattern at rest. PSS is a sensitive marker of stress-induced ischemia and extends often further than a visible wall motion abnormality. A single segment with PSS is therefore not likely to represent a relevant ischemic area. If a better understanding of the PSS extend is needed, or if an artefact has to be distinguished from true pathology, a strain rate curved M-mode is a helpful tool. The yellow and blue display of shortening and lengthening allows a visual distinction of the direction of deformation. While an ischemic zone can easily be identified by its temporal and spatial distribution, artefacts are recognized by the band-like pattern of inverted colours in one position throughout the cardiac cycle (Figure 8).

#### Summary and perspective

Current deformation imaging software is sufficiently developed to allow fast and reliable analysis and to provide clinically helpful answers. As any quantitative imaging technique, deformation imaging relies on the assumption that pathologic processes cause functional changes and that these changes can be visualized with sufficient accuracy. Improvements in both, tissue Doppler and speckle tracking algorithms would be desirable to reduce noise and improve measurement accuracy [11]. If further developments in 2D and 3D speckle tracking, such as layer specific strain, or if the assessment of all three normal strain components in 3D data sets results in an improved diagnostic value of echocardiographic examinations remains to be determined.

From a current perspective, strain and strain rate imaging by tissue Doppler and speckle tracking are helpful tools in the clinical assessment of CAD patients and should be part of every echocardiographic examination.
